# Comparative Effects of Different Dietary Acidifiers on Growth Performance and Intestinal Health in Weaned Piglets

**DOI:** 10.3390/ani16193103

**Published:** 2026-10-02

**Authors:** Zhiyuan Zhou, Yongtao Lai, Wen Ren, Anping Li, Bing Yu, Jun He, Hui Yan, Ping Zheng, Yueqi Xuan, Jie Yu

**Affiliations:** 1 Animal Nutrition Institute, Sichuan Agricultural University, Chengdu 611130, China; 2 Key Laboratory of Animal Disease-Resistant Nutrition, Ministry of Education, Chengdu 611130, China; 3 Animal Nutrition and Health, R&D Center, DSM-Firmenich, Bazhou 430023, China; 4 DSM China Co., Ltd., DSM-Firmenich, Shanghai 201203, China

**Keywords:** benzoic acid, permeabilizing complex, weaned piglets, growth performance, nutrient digestibility, intestinal morphology, fecal microbiota

## Abstract

This study evaluated the effects of conventional benzoic acid (BA), a benzoic acid formulation containing 97% benzoic acid and 3% Permeabilizing Complex™ (NBA), and a compound acidifier containing formic, acetic, propionic, and citric acids (CA) on growth performance, nutrient digestibility, intestinal morphology, serum parameters, and fecal microbiota in weaned piglets. A total of 128 piglets were assigned to four dietary treatments for 42 d: a basal diet without supplemental acidifier (CON), 0.5% BA, 0.3% NBA, or 0.3% CA. The acidifier products were evaluated at their respective manufacturer-recommended inclusion rates; therefore, the study represents a practical comparison of acidification strategies rather than an equal-benzoic-acid-dose comparison. BA increased final body weight (BW) compared with CON and increased average daily gain (ADG) during days 15–42 and 1–42 compared with CON and CA, while also increasing average daily feed intake (ADFI) compared with all other treatments (*p* < 0.05). NBA also increased ADG compared with CON (*p* < 0.05), with a numerical response intermediate between CON and BA. CA increased the apparent total tract digestibility of crude protein, ash, and gross energy compared with CON, BA, and NBA (*p* < 0.05), and increased dry matter digestibility compared with BA and NBA but not CON; however, these improvements were not accompanied by greater final BW or ADG. NBA increased serum IgM, whereas CA increased IgM and total antioxidant capacity but decreased IgG relative to CON (*p* < 0.05). Intestinal morphological responses were segment-specific. NBA increased duodenal villus height and villus height-to-crypt depth ratio and reduced ileal crypt depth while increasing the ileal ratio, but also increased jejunal crypt depth and reduced the jejunal ratio (*p* < 0.05). NBA and CA reduced fecal microbial richness, while dietary treatment significantly affected fecal microbial community membership. In conclusion, conventional BA produced the clearest feed intake and growth response, whereas NBA increased ADG relative to CON and produced segment-specific effects on intestinal morphology, and CA primarily increased apparent nutrient digestibility.

## Simple Summary

Weaning exposes piglets to nutritional, intestinal, and environmental challenges that can compromise feed intake and growth. Benzoic acid is commonly used in nursery diets, and formulations combining benzoic acid with other functional components are also available. This study compared conventional benzoic acid, a benzoic acid formulation containing a permeabilizing complex, and a compound acidifier in weaned piglets. Conventional benzoic acid produced the clearest feed-intake and growth response. The benzoic acid formulation containing the permeabilizing complex increased average daily gain compared with the control and produced segment-specific changes in intestinal morphology. In contrast, the compound acidifier improved nutrient digestibility, but this response did not translate into greater growth. Overall, conventional benzoic acid produced the clearest growth response, while the benzoic acid formulation increased average daily gain relative to the control and showed segment-specific effects on intestinal morphology.

## 1. Introduction

Weaning is a critical period in pig production, during which immature gastrointestinal function and abrupt dietary and environmental changes can impair nutrient utilization, intestinal homeostasis, and growth performance [1]. To mitigate these challenges, dietary organic acids have been widely used as feed additives to support growth performance and intestinal health in nursery pigs [2]. Among these additives, benzoic acid (BA) is one of the most extensively studied organic acids in nursery pig nutrition. Its practical relevance is supported by evidence of increased feed intake and growth, together with potential effects on nutrient utilization, intestinal morphology, and microbial composition, although responses depend on dose, diet composition, health status, and experimental conditions [3].

Formulation strategies have been developed to enhance the antimicrobial activity of organic acids against Gram-negative bacteria. One such approach incorporates a permeabilizing complex designed to increase the susceptibility of the bacterial outer membrane to organic acids and other antimicrobial compounds. Previous studies evaluating multicomponent formulations containing organic acids, phytogenic compounds, and a permeabilizing complex reported effects on growth performance and bacterial responses in weaned pigs challenged with enterotoxigenic *Escherichia coli* and in pigs exposed to *Salmonella* under field conditions [4,5]. However, these studies evaluated blended acidifier products, and information regarding a BA formulation containing a permeabilizing complex remains limited. Therefore, the effects of this formulation on growth performance, nutrient utilization, intestinal morphology, immune-related indices, and fecal microbiota require direct evaluation. Compound acidifiers containing blends of organic acids represent another commonly used dietary acidification strategy [6,7]. Because their acid composition, dissociation characteristics, buffering effects, and sites of activity may differ from those of BA-based products, compound acidifiers may produce different patterns of response in nutrient digestibility, intestinal physiology, and growth performance. However, few studies have compared conventional BA, a BA formulation containing a permeabilizing complex, and a compound acidifier within the same experiment using consistent diets, animal populations, and management conditions.

Therefore, the present study compared conventional BA, a BA formulation containing a permeabilizing complex, and a compound acidifier at their respective recommended inclusion rates in weaned piglets. We hypothesized that these acidification strategies would produce distinct responses in growth performance, intestinal morphology, and fecal microbial ecology.

## 2. Materials and Methods

### 2.1. Animal Ethics Statement

The experimental protocol was reviewed and approved by the Institutional Animal Care and Use Committee of Sichuan Agricultural University (approval number: 2023314111; approval date: 24 December 2023).

### 2.2. Animals, Experimental Design, and Diets

A total of 128 healthy Duroc × (Landrace × Yorkshire) piglets weaned at 21 d of age, with an initial body weight (BW) of 6.80 ± 0.47 kg, were used in a 42-d feeding trial. Piglets were randomly allocated according to initial body weight and gender to four dietary treatments, with eight replicate pens per treatment and four piglets per pen. The acidifier products used in this study were provided by DSM-Firmenich (Kaiseraugst, Switzerland), and their dietary inclusion levels were selected according to the manufacturer’s recommended supplementation rates. The four dietary treatments were as follows: (1) a basal diet without supplemental acidifier (CON); (2) the basal diet supplemented with 0.5% conventional benzoic acid (VevoVitall^®^, BA); (3) the basal diet supplemented with 0.3% of a benzoic acid formulation containing a permeabilizing complex (VevoVitall^®^ 2.0, NBA); and (4) the basal diet supplemented with 0.3% of a compound acidifier (CA). The conventional BA product contained 99.99% benzoic acid. The NBA product contained 97% benzoic acid and 3% Permeabilizing Complex™. The detailed composition of the Permeabilizing Complex™ is proprietary and was not disclosed by the manufacturer. The CA product was a compound acidifier containing formic acid (≥25%), acetic acid (≥7%), propionic acid (≥3%), and citric acid (≥5%), with silicon dioxide as a carrier, according to the manufacturer’s guaranteed analysis. All acidifier products were provided by DSM-Firmenich and were evaluated at their respective manufacturer-recommended dietary inclusion rates. Therefore, the experiment was designed as a practical comparison of acidification strategies rather than as a comparison based on equal product inclusion or active benzoic acid concentrations.

The experiment consisted of two feeding phases: days 1–14 and days 15–42. The basal diets were formulated to meet or exceed the nutrient requirements of weaned pigs according to NRC (2012) [8]. Separate diets were formulated for the 7–11 kg and 11–25 kg body-weight phases. The ingredient composition and nutrient levels of the basal diets are presented in Table 1. Each acidifier product replaced an equivalent amount of corn in the corresponding basal diet to maintain a constant total dietary inclusion of 100%. Piglets had ad libitum access to their respective experimental diets and drinking water throughout the trial.

### 2.3. Housing and Management

The experiment was conducted at the Teaching and Research Experimental Base, Animal Nutrition Institute, Sichuan Agricultural University. Piglets had free access to feed and water throughout the experiment. The room temperature was maintained at 25–28 °C, and relative humidity was maintained below 80%. Feed was replenished four times daily at 08:00, 12:00, 16:00, and 20:00. Pens were cleaned daily, and routine ventilation and disinfection procedures were applied throughout the experiment.

### 2.4. Sample Collection

#### 2.4.1. Feed and Fecal Samples for Nutrient Digestibility

Representative feed samples were collected from each dietary treatment using the quartering method, with approximately 250 g retained and stored at 4 °C for subsequent nutrient analysis. During days 39–42 of the experimental period, freshly voided fecal samples were collected twice daily, at approximately 12:20 and 20:20, from six predetermined pens per treatment, excluding the first and last pens. Fresh feces were collected promptly after defecation, with care taken to avoid visible contamination with feed, urine, or other foreign material. Samples collected within each selected pen on the same day were pooled. To minimize nitrogen loss and sample deterioration, 10 mL of 10% H_2_SO_4_ solution per 100 g of fresh feces and a small amount of toluene were added and thoroughly mixed with the samples. The daily pooled samples were stored at −20 °C. At the end of the 4-d collection period, fecal samples collected from the same pen across the four days were thawed, pooled, and thoroughly homogenized to form one composite sample. Composite samples were dried at 65 °C for 72 h, ground, and passed through a 40-mesh screen before chemical analysis. Each selected pen was considered one experimental unit.

#### 2.4.2. Blood Sampling

On day 43, one pig with a body weight close to the mean body weight of its respective pen was selected from each pen after a 12 h fast. This selection criterion was intended to select an individual representative of the average body weight of each pen. Approximately 10 mL of blood was collected from the anterior vena cava. Blood samples were allowed to clot at room temperature for 30 min and then centrifuged at 3000 rpm for 15 min at 4 °C. The resulting serum was collected, aliquoted, and stored at −20 °C until analysis.

#### 2.4.3. Fecal Samples for Microbiota Analysis

On day 43, one pig with a body weight close to the mean body weight of its respective pen was selected from each pen for fecal microbiota sampling. Fresh rectal fecal samples were aseptically collected from the selected pigs, immediately frozen in liquid nitrogen, and subsequently stored at −80 °C until 16S rRNA gene sequencing. One fecal sample was obtained from each pen, resulting in eight biological samples per treatment for microbial analysis.

#### 2.4.4. Intestinal Tissue Sampling

For intestinal tissue sampling, one pig with a body weight close to the mean body weight of its respective pen was selected from each of the same six predetermined pens per treatment, excluding the first and last pens. Following slaughter, approximately 2 cm segments were collected from the mid-portions of the duodenum, jejunum, and ileum and fixed in 4% paraformaldehyde for morphological analysis.

### 2.5. Growth Performance and Diarrhea Rate

Piglet body weights recorded at the beginning of the experiment and on days 14 and 42 were used to evaluate growth performance, and feed intake was recorded daily on a pen basis. Average daily feed intake (ADFI), average daily gain (ADG), and feed-to-gain ratio (F/G) were calculated for days 1–14, days 15–42, and the overall experimental period.

Fecal consistency was monitored throughout the experiment using a four-point scoring system: 0, normal, firm feces; 1, soft but formed feces; 2, unformed or semi-liquid feces; and 3, watery feces. Piglets with a fecal score ≥ 2 were considered to have diarrhea. Diarrhea rate was calculated as follows: diarrhea rate (%) = Σ (number of diarrheic piglets × number of diarrhea days)/(number of piglets × number of experimental days) × 100. The pen was considered the experimental unit for diarrhea rate.

### 2.6. Apparent Total Tract Digestibility

Apparent total tract digestibility (ATTD) of nutrients was determined from fecal samples collected during days 39–42 using acid-insoluble ash (AIA) as an internal marker; therefore, the resulting ATTD values represent nutrient digestibility during the terminal 4-d collection period rather than across the entire 42-d feeding trial. The AIA contents in the diets and fecal samples were determined according to the Chinese National Standard GB/T 23742 [9]. The contents of dry matter (DM), crude protein (CP), ether extract (EE), ash, and gross energy (GE) in the diets and fecal samples were determined according to the methods described by Zhang [10].

Apparent total tract digestibility was calculated according to the following equation:

$$\mathrm{ATTD}\left(\%\right)=100-100\times \frac{A_{1}F_{2}}{A_{2}F_{1}}$$

where $F_{1}$ and $F_{2}$ represent the concentrations of the corresponding nutrient in the diet and feces, respectively, and $A_{1}$ and $A_{2}$ represent the concentrations of AIA in the diet and feces, respectively.

### 2.7. Serum Immunological and Antioxidant Indices

Serum concentrations of immunoglobulin A (IgA), immunoglobulin M (IgM), and immunoglobulin G (IgG) were determined using commercial enzyme-linked immunosorbent assay (ELISA) kits (Jiangsu Meimian Industrial Co., Ltd., Yancheng, China) according to the manufacturer’s instructions. Serum total antioxidant capacity (T-AOC), total superoxide dismutase (T-SOD) activity, glutathione peroxidase (GSH-Px) activity, malondialdehyde (MDA) concentration, and catalase (CAT) activity were measured using commercial assay kits (Nanjing Jiancheng Bioengineering Institute, Nanjing, China) according to the manufacturer’s instructions.

### 2.8. Intestinal Morphology

Duodenal, jejunal, and ileal tissue samples fixed in 4% paraformaldehyde were dehydrated, cleared, embedded in paraffin, sectioned, and stained with hematoxylin and eosin. For each pig, one representative H&E-stained section from each intestinal segment was used for morphometric analysis. Histological sections were examined using an Olympus CX-31 light microscope (Olympus Corporation, Tokyo, Japan), and digital slide images were acquired using a NanoZoomer^®^ S360 digital slide scanner (Hamamatsu Photonics K.K., Hamamatsu, Japan). For each section, 15 intact, well-oriented, and spatially separated villi and their associated crypts were randomly selected for morphometric analysis. Measurements were performed under blinded conditions using NDP.view2 software (version 2.9.29; Hamamatsu Photonics K.K., Hamamatsu, Japan). Villus height was measured from the tip of the villus to the villus–crypt junction, and crypt depth was measured from the villus–crypt junction to the base of the crypt. The mean villus height and mean crypt depth of the 15 measurements were calculated for each intestinal segment of each pig. The villus height-to-crypt depth ratio (V/C) was subsequently calculated as the ratio of mean villus height to mean crypt depth.

### 2.9. Fecal Microbiota Analysis

Microbial genomic DNA was extracted from rectal fecal samples using the E.Z.N.A.^®^ Stool DNA Kit (Omega Bio-tek, Norcross, GA, USA) according to the manufacturer’s instructions. The V3–V4 region of the bacterial 16S rRNA gene was amplified using primers 341F (5′-CCTAYGGGRBGCASCAG-3′) and 806R (5′-GGACTACNNGGGTATCTAAT-3′). PCR amplification was performed in 20 μL reactions using TransStart FastPfu DNA Polymerase (TransGen Biotech, Beijing, China) under the following conditions: initial denaturation at 95 °C for 5 min; 28 cycles of 95 °C for 30 s, 55 °C for 30 s, and 72 °C for 45 s; and a final extension at 72 °C for 10 min. PCR products were purified, quantified, and used for library construction. Paired-end sequencing (2 × 300 bp) was performed on a DNBSEQ-G99 platform (MGI Tech Co., Ltd., Shenzhen, China) by Shanghai BIOZERON Biotechnology Co., Ltd. (Shanghai, China). Across the 32 libraries, 50,373–69,821 raw read pairs were generated per sample (mean, 61,127).

Raw paired-end reads were quality-filtered using Trimmomatic v0.36. Reads were truncated when the average quality score within a 10-bp sliding window was below 20, and reads shorter than 50 bp after trimming were discarded. Paired-end reads were merged using FLASH v1.2.7, with a minimum overlap of 10 bp and a maximum mismatch ratio of 0.2 within the overlapping region. Sequences were demultiplexed based on exact barcode matching, with up to two mismatches allowed in primer matching. Chimeric sequences were identified and removed using USEARCH against the Gold reference database in combination with de novo chimera detection. High-quality sequences were clustered into operational taxonomic units (OTUs) at 97% sequence similarity using UPARSE/USEARCH v10. Taxonomic assignment was performed using the UCLUST algorithm against the SILVA 138.1 database with a confidence threshold of 0.8. The OTU table was rarefied to 49,850 sequences per sample before downstream diversity and community-composition analyses.

Alpha diversity was evaluated using the Chao1 richness and Shannon diversity indices. Differences in alpha-diversity indices among treatments were assessed using the Kruskal–Wallis test. When a significant overall treatment effect was detected, pairwise comparisons were performed using Dunn’s test with Holm adjustment for multiple comparisons. Beta diversity was evaluated by principal coordinate analysis (PCoA) based on unweighted and weighted UniFrac distance matrices. Differences in overall microbial community structure among treatments were assessed by permutational multivariate analysis of variance (PERMANOVA) using the adonis2 function in the vegan package (version 2.7-6) in R (version 4.5.3), with 9999 permutations. Homogeneity of multivariate dispersion among treatments was assessed using the betadisper function followed by permutest, also with 9999 permutations.

Differential microbial taxa among treatments were identified using linear discriminant analysis effect size (LEfSe) [11]. The Kruskal–Wallis rank-sum test was used for initial screening of differential taxa, followed by linear discriminant analysis to estimate the effect size of each discriminative taxon. Taxa with *p* < 0.05 and an LDA score > 2.0 were considered discriminative. No additional false discovery rate adjustment was applied within the original LEfSe workflow. As a sensitivity analysis, genus-level *p*-values were additionally adjusted using the Benjamini–Hochberg procedure, with *q* < 0.05 considered statistically significant.

### 2.10. Statistical Analysis

Non-microbial data were initially organized using Microsoft Excel 2019 and subsequently analyzed using SPSS Statistics 27.0 (IBM Corp., Armonk, NY, USA). The pen was considered the experimental unit. The experimental design included eight replicate pens per treatment, and no formal a priori power calculation was performed. All eight pens per treatment were included for growth performance and diarrhea rate. Six predetermined pens per treatment, excluding the first and last pens, were used for apparent total tract digestibility and intestinal morphology analyses. For serum indices, one pig from each of the eight pens was sampled to represent the pen. Analyses were performed using the available observations for each endpoint, and missing observations were not imputed.

Body weight measured on days 0, 14, and 42 was analyzed using a repeated-measures linear mixed model, with treatment, time, and the treatment × time interaction included as fixed effects and pen specified as the repeated-measures subject. Compound symmetry (CS), first-order autoregressive [AR(1)], and unstructured (UN) covariance structures were evaluated, and the covariance structure with the best model fit based on AIC, BIC, and model convergence was used in the final analysis. An unstructured covariance structure was used for BW. When the treatment × time interaction was significant, treatment effects were examined within each time point using Tukey-adjusted pairwise comparisons.

ADG, ADFI, F/G, diarrhea rate, apparent total tract digestibility, serum indices, and intestinal morphology endpoints were analyzed for their predefined periods or sampling time points using one-way analysis of variance (ANOVA). Model residuals were assessed for normality using the Shapiro–Wilk test, and homogeneity of variances was assessed using Levene’s test. When the overall treatment effect was significant (*p* < 0.05), treatment means were compared using Duncan’s multiple range test. Potential outliers were screened within each treatment for each endpoint using the boxplot procedure in the Explore function of SPSS. Mild outliers, defined as observations located 1.5–3.0 interquartile ranges (IQRs) beyond the lower or upper quartile, were retained, whereas extreme values located more than 3.0 IQRs beyond the lower or upper quartile were excluded. The same criterion was applied consistently across treatments and endpoints. Data are presented as means with pooled standard error of the mean (SEM). For endpoints with unequal sample sizes, pooled SEM was calculated from the residual mean square using the harmonic mean of the treatment sample sizes. Statistical analyses of fecal microbiota data were performed as described in Section 2.9.

## 3. Results

### 3.1. Growth Performance and Diarrhea Rate

The effects of dietary acidifiers on growth performance and diarrhea rate are presented in Table 2. Repeated-measures analysis of BW revealed a significant effect of time (*p* < 0.001) and a significant treatment × time interaction (*p* = 0.027), whereas the overall treatment effect was not significant (*p* = 0.127). No treatment differences in BW were detected on days 0 or 14, whereas a treatment effect was observed on day 42 (*p* = 0.027). On day 42, BA pigs had greater BW than CON pigs (Tukey-adjusted *p* < 0.05). Dietary treatment also affected ADFI and ADG during days 15–42 and 1–42 (*p* < 0.05). Compared with CON, BA increased ADFI and ADG during days 15–42 and 1–42, whereas NBA increased ADG during these periods (*p* < 0.05). Among the acidifier treatments, BA resulted in greater ADG than CA and greater ADFI than NBA and CA during days 15–42 and 1–42 (*p* < 0.05). No significant treatment effects were observed for F/G or diarrhea rate (*p* > 0.05).

### 3.2. Apparent Total Tract Digestibility

The effects of dietary acidifiers on the apparent total tract digestibility (ATTD) of nutrients are presented in Table 3. Compared with CON, BA and NBA did not significantly affect the ATTD of CP, ash, EE, DM, or GE (*p* > 0.05). CA increased the ATTD of CP, ash, and GE compared with CON and both BA-based treatments (*p* < 0.05). For DM, CA had greater ATTD than BA and NBA (*p* < 0.05) but did not differ significantly from CON (*p* > 0.05). For ash digestibility, BA was greater than NBA (*p* < 0.05), although neither BA nor NBA differed significantly from CON (*p* > 0.05). No significant treatment effect was observed for EE (*p* > 0.05).

### 3.3. Serum Immunological and Antioxidant Indices

The effects of dietary acidifiers on serum immunological and antioxidant indices are presented in Table 4. Compared with CON, BA did not significantly affect the measured serum indices, NBA increased IgM, and CA increased IgM and T-AOC but reduced IgG (*p* < 0.05). Among the acidifier treatments, CA had a higher IgM concentration than BA and NBA, while NBA had a higher IgM concentration than BA (*p* < 0.05). CA also had lower IgG than BA and NBA and higher T-AOC than NBA (*p* < 0.05). No significant treatment effects were observed for IgA, T-SOD, GSH-Px, MDA, or CAT (*p* > 0.05).

### 3.4. Intestinal Morphology

The effects of dietary acidifiers on intestinal morphology are presented in Table 5. Dietary acidifier effects varied among intestinal segments. In the duodenum, NBA increased villus height and V/C ratio compared with CON, whereas CA increased crypt depth and reduced the V/C ratio (*p* < 0.05). Among the acidifier treatments, NBA had greater villus height and V/C ratio than BA and CA, whereas CA had greater crypt depth than BA and NBA (*p* < 0.05). In the jejunum, NBA increased crypt depth compared with CON, while all acidifier treatments reduced the V/C ratio relative to CON (*p* < 0.05). NBA also had greater crypt depth than BA (*p* < 0.05), whereas villus height did not differ among treatments (*p* > 0.05). In the ileum, NBA and CA reduced crypt depth and increased the V/C ratio compared with CON (*p* < 0.05). Among the acidifier treatments, NBA had lower crypt depth than CA and BA, while CA had lower crypt depth than BA; both NBA and CA had greater V/C ratios than BA (*p* < 0.05). Ileal villus height did not differ among treatments (*p* > 0.05).

### 3.5. Fecal Microbiota

**Figure 1:**
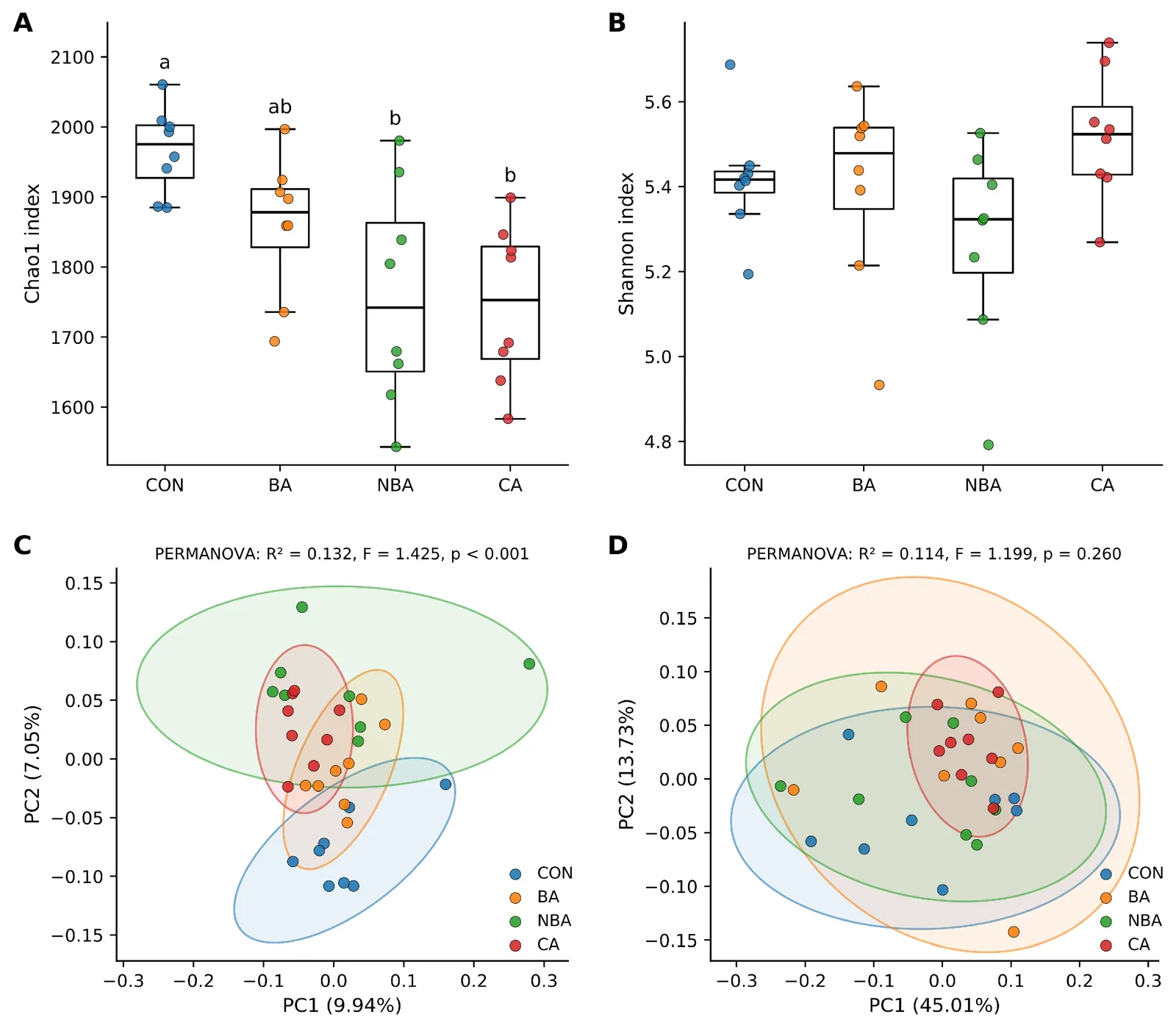
Effects of dietary acidifiers on fecal microbial diversity and community structure in weaned piglets. (**A**) Chao1 index. (**B**) Shannon index. (**C**) Principal coordinate analysis based on unweighted UniFrac distances. (**D**) Principal coordinate analysis based on weighted UniFrac distances. Differences in microbial community structure were evaluated using PERMANOVA. Different lowercase letters in the alpha-diversity panels indicate significant pairwise differences based on Dunn’s test with Holm adjustment (adjusted *p* < 0.05). Ellipses represent 95% covariance ellipses calculated from the within-group distributions of PC1 and PC2 scores. Each treatment included eight biological replicates (*n* = 8).

The effects of dietary acidifiers on fecal microbial diversity and community structure are shown in Figure 1. The Chao1 index differed among treatments (Kruskal–Wallis, *p* = 0.002). Pairwise comparisons using Dunn’s test with Holm adjustment showed lower Chao1 richness in the NBA and CA groups than in the CON group (adjusted *p* = 0.009 and 0.004, respectively), whereas BA did not differ significantly from CON (adjusted *p* > 0.05; Figure 1A). The Shannon index did not differ among treatments (Kruskal–Wallis, *p* = 0.094; Figure 1B). Principal coordinate analysis based on unweighted UniFrac distances showed a significant treatment effect on fecal microbial community structure (PERMANOVA, R^2^ = 0.132, F = 1.425, *p* < 0.001; Figure 1C). In contrast, no significant treatment effect was detected based on weighted UniFrac distances (PERMANOVA, R^2^ = 0.114, F = 1.199, *p* = 0.260; Figure 1D). Multivariate dispersion did not differ significantly among treatments for either unweighted or weighted UniFrac distances (PERMDISP, F = 2.414, *p* = 0.058 and F = 1.674, *p* = 0.198, respectively).

**Figure 2:**
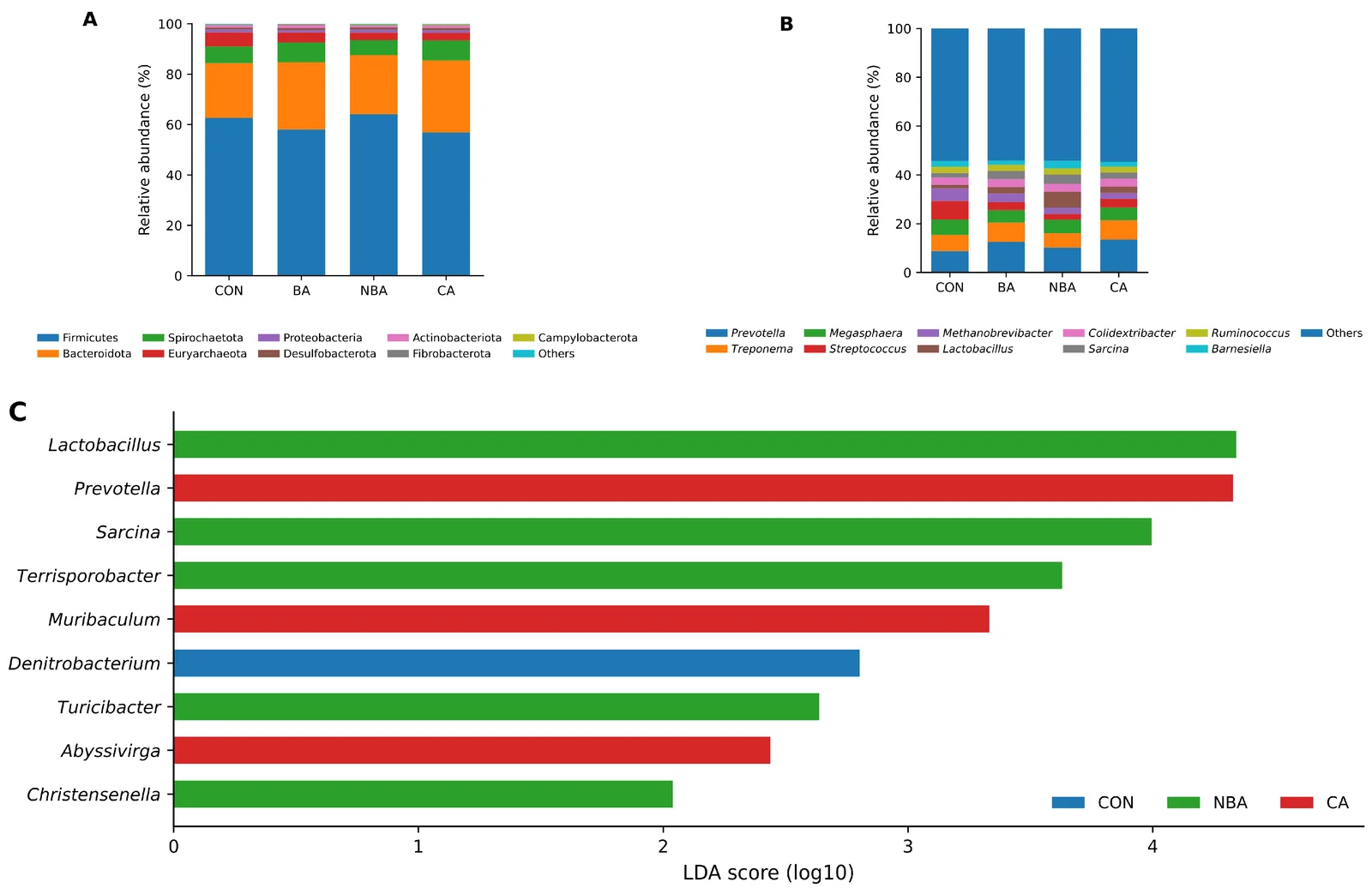
Effects of dietary acidifiers on fecal microbial composition and discriminative taxa in weaned piglets. (**A**) Relative abundance of predominant microbial phyla. (**B**) Relative abundance of predominant microbial genera. (**C**) Genus-level discriminative taxa identified by LEfSe using a Kruskal–Wallis test (*p* < 0.05) and an LDA score threshold > 2.0. No FDR correction was applied in the original LEfSe workflow.

At the phylum level, Firmicutes and Bacteroidota were the predominant microbial taxa across all treatments, followed by Spirochaetota and Euryarchaeota (Figure 2A). At the genus level, *Prevotella*, *Treponema*, *Megasphaera*, *Streptococcus*, *Methanobrevibacter*, *Lactobacillus*, *Colidextribacter*, *Sarcina*, and *Ruminococcus* were among the predominant genera (Figure 2B). Genus-level LEfSe analysis identified *Denitrobacterium* as a discriminative taxon associated with the CON group; *Lactobacillus*, *Sarcina*, *Terrisporobacter*, *Turicibacter*, and *Christensenella* with the NBA group; and *Prevotella*, *Muribaculum*, and *Abyssivirga* with the CA group (Figure 2C). In a complementary genus-level sensitivity analysis applying Benjamini–Hochberg correction across all tested genera, no taxa remained significant at *q* < 0.05. No genus-level discriminative taxon was identified for the BA group under the applied LEfSe criteria.

## 4. Discussion

The principal finding of this study was that the three acidifier products produced distinct response profiles across growth performance and physiological outcomes. Conventional BA produced the clearest growth response, characterized by greater final BW than CON and greater ADG than CON and CA, together with greater feed intake, whereas NBA increased ADG relative to CON and showed intermediate numerical values for final BW and ADG. In contrast, CA produced the strongest response in apparent nutrient digestibility but did not significantly improve final BW or overall ADG relative to CON. These findings indicate that acidifier efficacy should be evaluated across both production and intestinal outcomes rather than inferred from a single physiological response.

Dietary BA at approximately 0.5–0.6% has repeatedly been associated with improved growth performance in nursery pigs [3,12,13,14], and a recent quantitative synthesis estimated an optimal inclusion level of approximately 0.60% for ADG [3]. These findings are consistent with the pronounced growth response observed with 0.5% conventional BA in the present study. A previous study evaluating protected BA did not demonstrate a superior growth response compared with free BA [15], indicating that modification of BA formulation does not necessarily translate into greater growth performance. In the present study, conventional BA and NBA were supplied at their manufacturer-recommended inclusion levels of 0.5% and 0.3%, respectively; therefore, differences between them cannot be attributed solely to formulation. The response to conventional BA was accompanied by greater feed intake, suggesting that increased voluntary feed intake contributed, at least in part, to the greater ADG. This interpretation is supported by previous work showing a dose-dependent increase in feed intake and body-weight gain in piglets supplemented with BA [12]. NBA did not significantly increase ADFI relative to CON but nevertheless increased ADG relative to CON and showed intermediate numerical values for final BW and ADG. Because the products differed in inclusion rate and luminal BA exposure was not measured, the mechanisms underlying these different response patterns cannot be established from the present study.

The intestinal morphology results further showed that the effects of NBA were segment-specific. NBA increased duodenal villus height and V/C ratio and reduced ileal crypt depth while increasing the ileal V/C ratio. Previous studies using conventional BA have also reported changes in intestinal morphology in weaned pigs. Halas et al. reported increased villus height following supplementation with 0.5% BA, whereas Diao et al. observed increased villus height and V/C ratios and reduced crypt depth in selected intestinal segments of piglets fed 0.5% BA [16,17]. In contrast, a previous study comparing free and protected BA detected no significant treatment effects on jejunal or ileal villus height, crypt depth, or villus height-to-crypt depth ratio [15], indicating that morphological responses to BA supplementation are not necessarily consistent across studies. Villus height, crypt depth, and the V/C ratio are commonly used as indicators of intestinal mucosal architecture [18]; however, these morphological indices alone do not directly demonstrate improved absorptive or barrier function. In the present study, NBA also increased jejunal crypt depth and reduced the jejunal V/C ratio relative to CON. CA similarly produced opposite responses among intestinal segments, with greater crypt depth and a lower V/C ratio in the duodenum but lower crypt depth and a greater V/C ratio in the ileum. Thus, the effects of both NBA and CA should be interpreted as localized, segment-specific morphological responses rather than as uniform improvement throughout the small intestine.

CA increased the ATTD of CP, ash, and GE relative to CON and both BA-based treatments, whereas DM digestibility was greater than that in the BA and NBA groups but did not differ significantly from CON. Previous studies have reported improvements in nutrient digestibility following supplementation with mixed or compound organic acids, although the magnitude and affected nutrients have varied among formulations. Long et al. reported greater apparent total tract digestibility of several nutrients in weaned piglets supplemented with mixed organic acids, whereas Li et al. observed increased crude protein digestibility following supplementation with a compound organic acid [6,19]. Xu et al. likewise reported improvements in apparent total tract nutrient digestibility attributable to organic-acid supplementation [20]. In contrast, Lee et al., using mixtures containing formic and propionic acids, detected no significant effects on the apparent total tract digestibility of DM, GE, or CP [7]. Together, these findings indicate that the digestibility response to dietary acidification is not uniform across organic-acid formulations and experimental conditions. In the present study, the greater terminal ATTD observed with CA was not accompanied by greater feed intake, final BW, ADG, or feed efficiency. An improvement in apparent nutrient digestibility does not necessarily imply a proportional increase in nutrient retention or growth, and previous studies have shown that digestibility and growth responses to dietary acidification do not always occur in parallel [12,21]. In addition, responses to organic-acid supplementation may depend on characteristics of the basal diet. Li et al. reported limited growth responses to organic-acid supplementation in pigs fed a highly digestible diet, whereas more pronounced responses were observed under less digestible dietary conditions [22]. Finally, ATTD in the present study was determined from fecal samples collected only during the final four days of the 42-d experiment, whereas growth performance reflected cumulative responses across the entire feeding period. Therefore, the greater digestibility observed near the end of the trial cannot be assumed to have occurred to the same extent throughout the experiment. Together, these considerations indicate that greater terminal ATTD alone should not be interpreted as evidence of superior overall biological efficacy in the absence of corresponding production responses.

The serum responses were limited to a small number of variables. NBA increased serum IgM compared with CON and BA, whereas CA produced the highest IgM concentration and greater T-AOC than CON and NBA but reduced IgG compared with all other treatments. Previous studies have also reported changes in selected immune- and antioxidant-related indices following supplementation with mixed or compound organic acids. Long et al. reported tendencies toward higher serum IgG and T-AOC, together with a reduction in serum hydroxyl radicals, in piglets receiving one of the mixed organic-acid formulations [6]. Li et al. similarly observed increased serum T-AOC and altered IL-10 concentrations following compound organic-acid supplementation [19]. These findings indicate that dietary organic-acid supplementation can influence selected systemic immune- and antioxidant-related indices, although the direction and magnitude of the responses vary among formulations and experimental conditions. Accordingly, the changes observed in the present study should be interpreted as modulation of individual serum indices rather than as evidence of a generalized enhancement of systemic immunity or antioxidant capacity.

Fecal microbiota responses were treatment-dependent. NBA and CA were associated with lower Chao1 richness than CON, whereas Shannon diversity did not differ among treatments. Treatment affected unweighted but not weighted UniFrac community structure. Because unweighted UniFrac emphasizes differences in community membership, whereas weighted UniFrac additionally incorporates the relative abundance of taxa, this discordance suggests that treatment-associated separation was more evident in the presence or absence of phylogenetic lineages than in abundance-weighted community structure [23]. However, this interpretation should remain cautious because multivariate dispersion for unweighted UniFrac approached, but did not reach, statistical significance. Previous studies in pigs have also shown that dietary benzoic acid or organic-acid combinations can alter intestinal or fecal microbial composition, although the affected taxa and direction of change vary among formulations and experimental conditions [17,24,25]. In the present study, several genera, including Lactobacillus, were identified by LEfSe as exploratory discriminative taxa associated with NBA. However, none of the genus-level associations remained significant after Benjamini–Hochberg correction in the complementary sensitivity analysis; therefore, these findings should be interpreted cautiously and should not be taken as evidence of treatment-specific functional effects. Reduced richness should likewise not be interpreted by itself as improved intestinal health. Furthermore, fecal microbiota cannot be assumed to represent microbial communities in the small intestine. Previous studies have demonstrated marked differences in microbial composition among gastrointestinal regions and between digesta- and mucosa-associated communities in pigs [26,27]. Accordingly, the fecal microbiota data in the present study cannot directly explain the morphological responses observed in the duodenum, jejunum, or ileum.

One limitation of the present study is that pigs selected for blood, fecal microbiota, and intestinal sampling were chosen based on proximity to the mean body weight of each pen rather than by random animal-level sampling. This approach was intended to obtain animals representative of the average body weight of each pen, but it may have underrepresented pigs at the extremes of the within-pen body-weight distribution. Therefore, the serum, microbiota, and intestinal measurements should be interpreted as responses of pigs representative of the average pen body weight rather than as estimates of the full range of individual-level variability.

Previous evidence indicates that responses to dietary acidification are influenced not only by the acidifier itself but also by characteristics of the basal diet and the overall acidification strategy [22,28]. Accordingly, the present study should be interpreted as a practical comparison of three commercial acidification strategies evaluated at their respective manufacturer-recommended inclusion rates, rather than as an equal-dose comparison or a mechanistic test of formulation technology. From a practical perspective, based on supplier-provided reference prices and the inclusion rates used in the present study, the approximate additive costs of BA and NBA were similar on a per-ton-of-feed basis, whereas that of CA was somewhat lower. However, total feed costs, revenue from additional weight gain, and other production costs were not systematically evaluated; therefore, no formal conclusions regarding cost-effectiveness or return on investment can be drawn.

## 5. Conclusions

Under the manufacturer-recommended inclusion rates evaluated in the present study, the three acidification strategies produced distinct response profiles. Conventional BA at 0.5% yielded the clearest growth response, characterized by increased feed intake, final BW, and ADG. NBA at 0.3% increased ADG versus the control and produced segment-specific changes in intestinal morphology, whereas CA at 0.3% increased the apparent total tract digestibility of several nutrients without a corresponding improvement in growth performance. Because the products differed in composition and inclusion rate, these findings should be interpreted as a practical comparison of dietary acidification strategies rather than evidence of formulation-specific superiority.

## Figures and Tables

**Table 1:** Ingredient composition and nutrient levels of the basal diets (air-dry basis).

Item	7–11 kg	11–25 kg
Ingredient, %		
Corn, CP 7.8%	24.1	31.73
Extruded corn, CP 7.8%	28.1	30.20
Soybean meal, CP 46%	7.10	9.80
Extruded soybean	7.30	6.80
Broken rice	6.90	–
Soybean protein concentrate	7.80	6.00
Fish meal, CP 62.5%	2.70	2.50
Whey powder, CP 3%	6.20	5.00
Sucrose	2.00	2.00
Soybean oil	4.00	2.50
Choline chloride	0.10	0.10
Sodium chloride	0.30	0.30
Limestone	1.11	1.10
Dicalcium phosphate	0.70	0.60
L-Lysine HCl, 98%	0.58	0.53
DL-Methionine, 99%	0.25	0.18
L-Threonine, 98.5%	0.26	0.18
L-Tryptophan, 98.5%	0.05	0.03
Vitamin premix ^1^	0.05	0.05
Mineral premix ^2^	0.40	0.40
Total	100	100
Nutrient levels ^3^		
Digestible energy, MJ/kg	14.82	14.86
Crude protein, %	18.66	18.25
Calcium, %	0.85	0.80
Total phosphorus, %	0.58	0.55
Available phosphorus, %	0.39	0.36
SID lysine, %	1.41	1.35
SID methionine, %	0.54	0.47
SID cysteine, %	0.28	0.27
SID methionine + cystine, %	0.82	0.74
SID threonine, %	0.88	0.79
SID tryptophan, %	0.25	0.22

^1^ The vitamin premix provided the following per kg of diet: vitamin A, 15,000 IU; vitamin D3, 5000 IU; vitamin E, 40 IU; vitamin K3, 5 mg; vitamin B1, 5 mg; vitamin B2, 12.5 mg; vitamin B6, 6 mg; vitamin B12, 0.06 mg; biotin, 0.25 mg; niacin, 50 mg; pantothenic acid, 25 mg; and folic acid, 2.5 mg. ^2^ The mineral premix provided the following per kg of diet: Fe, 100 mg as FeSO_4_·H_2_O; Cu, 6 mg as CuSO_4_·5H_2_O; Mn, 4 mg as MnSO_4_·H_2_O; I, 0.14 mg as KI; Se, 0.30 mg as Na_2_SeO_3_; and Zn, 100 mg as ZnSO_4_. ^3^ Nutrient levels were calculated values. SID, standardized ileal digestible.

**Table 2:** Effects of dietary acidifiers on growth performance and diarrhea rate of weaned piglets.

Item	CON	BA	NBA	CA	SEM	*p*-Value
Body weight, kg						
Day 0	6.80	6.81	6.80	6.80	0.17	1.000
Day 14	8.27	8.81	8.53	8.50	0.25	0.522
Day 42	17.49 ^b^	20.12 ^a^	19.30 ^ab^	17.93 ^ab^	0.64	0.027
ADFI, g/d						
Days 1–14	231.18	283.26	254.84	244.40	15.31	0.124
Days 15–42	672.09 ^b^	809.01 ^a^	723.08 ^b^	693.50 ^b^	23.39	0.002
Days 1–42	525.12 ^b^	633.76 ^a^	567.00 ^b^	543.80 ^b^	19.33	0.003
ADG, g/d						
Days 1–14	104.74	142.83	123.93	121.67	9.65	0.071
Days 15–42	330.12 ^c^	403.11 ^a^	381.85 ^ab^	336.50 ^bc^	16.67	0.011
Days 1–42	254.26 ^c^	316.95 ^a^	298.20 ^ab^	264.72 ^bc^	13.00	0.007
F/G						
Days 1–14	2.26	2.04	2.09	2.02	0.105	0.377
Days 15–42	2.05	2.02	1.91	2.07	0.053	0.152
Days 1–42	2.09	2.01	1.91	2.06	0.051	0.089
Diarrhea rate, %						
Days 1–14	18.08	18.75	17.63	24.11	3.07	0.425
Days 15–42	14.84	13.27	13.86	16.89	2.37	0.719
Days 1–42	16.21	15.17	15.09	19.21	2.28	0.551

Values are presented as means with pooled SEM. The pen was considered the experimental unit (*n* = 8 pens per treatment). For BW, treatment comparisons within each time point were based on the repeated-measures linear mixed model with Tukey adjustment. For ADFI, ADG, F/G, and diarrhea rate, different superscript letters within a row indicate significant differences among treatments according to Duncan’s multiple range test (*p* < 0.05).

**Table 3:** Effects of dietary acidifiers on apparent total tract digestibility of nutrients in weaned piglets.

Item, %	CON	BA	NBA	CA	SEM	*p*-Value
CP	79.80 ^b^	80.85 ^b^	80.13 ^b^	85.14 ^a^	1.20	0.015
Ash	57.27 ^bc^	60.13 ^b^	54.59 ^c^	68.63 ^a^	1.69	<0.001
EE	76.55	77.86	77.99	80.85	1.47	0.261
DM	88.04 ^ab^	86.44 ^b^	86.48 ^b^	90.39 ^a^	0.93	0.022
GE	87.73 ^b^	87.56 ^b^	86.52 ^b^	90.36 ^a^	0.75	0.010

Values are presented as means with pooled SEM. Six pens per treatment were designated for fecal collection, with the pen considered the experimental unit. The numbers of available observations for CON/BA/NBA/CA were 5/5/6/6 for CP, 4/5/5/6 for ash, 5/5/6/5 for EE, 6/6/6/6 for DM, and 6/5/6/6 for GE, respectively. Missing observations were not imputed, and analyses were performed using the available observations for each endpoint. Different superscript letters within a row indicate significant differences among treatments according to Duncan’s multiple range test (*p* < 0.05). CP, crude protein; EE, ether extract; DM, dry matter; GE, gross energy; SEM, standard error of the mean.

**Table 4:** Effects of dietary acidifiers on serum immunological and antioxidant indices in weaned piglets.

Item	CON	BA	NBA	CA	SEM	*p*-Value
IgA, μg/mL	21.09	19.40	23.64	23.17	1.40	0.142
IgM, μg/mL	25.34 ^c^	23.11 ^c^	28.97 ^b^	34.09 ^a^	1.13	<0.001
IgG, μg/mL	304.97 ^a^	321.98 ^a^	327.33 ^a^	243.29 ^b^	20.46	0.027
T-AOC, mM	1.13 ^b^	1.17 ^ab^	1.13 ^b^	1.21 ^a^	0.02	0.043
T-SOD, U/mL	88.64	97.86	108.51	94.45	5.71	0.118
GSH-Px, U/mL	1729.12	1473.39	1522.01	1659.72	110.96	0.348
MDA, nmol/mL	1.33	2.04	1.80	1.79	0.23	0.175
CAT, U/mL	9.18	9.07	8.61	8.99	0.96	0.977

Values are presented as means with pooled SEM. Eight pigs per treatment, one pig from each pen, were sampled. No serum observations met the predefined criterion for exclusion as an extreme value. One MDA observation in the CA group was missing; therefore, *n* = 7 for this treatment–endpoint combination and *n* = 8 otherwise. Different superscript letters within a row indicate significant differences among treatments according to Duncan’s multiple range test (*p* < 0.05). IgA, immunoglobulin A; IgM, immunoglobulin M; IgG, immunoglobulin G; T-AOC, total antioxidant capacity; T-SOD, total superoxide dismutase; GSH-Px, glutathione peroxidase; MDA, malondialdehyde; CAT, catalase; SEM, standard error of the mean.

**Table 5:** Effects of dietary acidifiers on intestinal morphology in weaned piglets.

Item	CON	BA	NBA	CA	SEM	*p*-Value
Duodenum						
Villus height, μm	341.27 ^b^	333.64 ^b^	433.11 ^a^	359.54 ^b^	19.78	0.011
Crypt depth, μm	73.24 ^b^	68.34 ^b^	65.14 ^b^	114.56 ^a^	6.51	<0.001
V/C	5.16 ^b^	4.89 ^b^	6.69 ^a^	3.13 ^c^	0.415	<0.001
Jejunum						
Villus height, μm	342.80	288.14	338.41	321.99	19.52	0.218
Crypt depth, μm	111.79 ^b^	116.60 ^b^	129.57 ^a^	119.07 ^ab^	4.14	0.042
V/C	3.09 ^a^	2.47 ^b^	2.60 ^b^	2.70 ^b^	0.129	0.017
Ileum						
Villus height, μm	283.91	285.55	244.90	278.40	13.31	0.138
Crypt depth, μm	112.97 ^a^	113.60 ^a^	75.65 ^c^	91.60 ^b^	3.78	<0.001
V/C	2.52 ^b^	2.51 ^b^	3.26 ^a^	3.04 ^a^	0.108	<0.001

Values are presented as means with pooled SEM (*n* = 6 pigs per treatment, except for duodenal morphology in the NBA group, *n* = 5 after exclusion of one extreme value). Different superscript letters within a row indicate significant differences among treatments according to Duncan’s multiple range test (*p* < 0.05). V/C, villus height-to-crypt depth ratio; SEM, standard error of the mean.

## Data Availability

The datasets supporting the findings of this study are available from the corresponding author upon reasonable request. The raw 16S rRNA gene sequencing data have been deposited in the NCBI Sequence Read Archive (SRA) under BioProject accession number PRJNA1525529.

## Abbreviations

The following abbreviations are used in this manuscript:

ADFI	Average daily feed intake
ADG	Average daily gain
ATTD	Apparent total tract digestibility
BA	Benzoic acid
CA	Compound acidifier
F/G	Feed-to-gain ratio
GSH-Px	Glutathione peroxidase
NBA	Benzoic acid formulation containing a permeabilizing complex
T-AOC	Total antioxidant capacity
T-SOD	Total superoxide dismutase
V/C	Villus height-to-crypt depth ratio
